# Combination of the ABL kinase inhibitor imatinib with the Janus kinase 2 inhibitor TG101348 for targeting residual BCR-ABL-positive cells

**DOI:** 10.1186/1756-8722-7-37

**Published:** 2014-04-28

**Authors:** Seiichi Okabe, Tetsuzo Tauchi, Seiichiro Katagiri, Yuko Tanaka, Kazuma Ohyashiki

**Affiliations:** 1 First Department of Internal Medicine, Tokyo Medical University, Shinjuku-ku, Tokyo 160-0023, Japan

**Keywords:** Chronic myeloid leukemia, JAK2 inhibitor, Imatinib, Cytokine, Feeder cell

## Abstract

**Background:**

The ABL kinase inhibitor imatinib is highly effective in treating most, but not all, patients with chronic myeloid leukemia (CML). This is because residual CML cells are generally present in the bone marrow microenvironment and are refractory to imatinib. Hematopoietic cytokine receptor signaling is mediated by Janus kinases (JAKs) and their downstream transcription factor, signal transducer and activator of transcription (STAT). TG101348 (SAR302503) is an oral inhibitor of JAK2.

**Methods:**

We investigated the efficacy of imatinib and TG101348 using the break point cluster region-c-Abelson (BCR-ABL)-positive cell line and primary CML samples wherein leukemia cells were protected by a feeder cell line (HS-5).

**Results:**

Imatinib treatment resulted in partial inhibition of cell growth in HS-5-conditioned medium. Furthermore, combined treatment with imatinib and TG101348 abrogated the protective effects of HS-5-conditioned medium on K562 cells. Phosphorylation of Crk-L, a BCR-ABL substrate, decreased considerably, while apoptosis increased. In addition, the combined treatment of CD34-positive primary samples resulted in considerably increased cytotoxicity, decreased Crk-L phosphorylation, and increased apoptosis. We also investigated TG101348 activity against feeder cells and observed that STAT5 phosphorylation, granulocyte macrophage colony-stimulating factor, and interleukin 6 levels decreased, indicating reduced cytokine production in HS-5 cells treated with TG101348.

**Conclusions:**

These results showed that JAK inhibitors may enhance the cytotoxic effect of imatinib against residual CML cells and that a combined approach may be a powerful strategy against the stroma-associated drug resistance of Philadelphia chromosome-positive cells.

## Background

Chronic myeloid leukemia (CML) is a malignant myeloproliferative clonal disorder associated with the presence of the Philadelphia chromosome (Ph) [1]. Ph results from a translocation that yields the break point cluster region-c-Abelson (BCR-ABL) fusion protein, which is a constitutively active tyrosine kinase [2]. BCR-ABL activation is responsible for modulating different signaling pathways, and consequently, hematopoietic stem and progenitor cell proliferation, as well as abnormal interactions with the extracellular matrix and stroma [3]. The BCR-ABL fusion protein activates several downstream signaling molecules, including those of the Ras/Raf/MAPK, PI3K/AKT/mTOR, and JAK/STAT pathways [4-6].

Imatinib, a first generation ABL tyrosine kinase inhibitor (ABL TKI), is a competitive inhibitor that binds to the ATP-binding catalytic site of ABL and decreases kinase domain activity by stabilizing the protein in an active conformation [7]. Imatinib is highly for treating patients in the chronic phase of CML. As observed in the International Randomized Study of Interferon and STI571 study, 87% of patients treated with imatinib achieved a complete cytogenetic response, with many patients achieving major molecular responses [8]. However, discontinuing imatinib is usually associated with disease relapse. In particular, the French Stop Imatinib trial reported that approximately 60% of patients who had maintained a complete molecular response for 2 years on imatinib relapsed after termination of treatment [9]. Thus, it is currently recommended that patients with CML stop TKI therapy only in the context of a clinical trial. The major cause of relapse in patients with CML is considered to be the existence of leukemia stem cells as imatinib is believed to be unable to kill quiescent CML stem cells. It is possible that these quiescent leukemia stem cells do not depend on BCR-ABL signaling [10]. Therefore, new strategies are required to specifically target CML stem cells and improve outcomes in patients with CML.

Janus kinases (JAKs) are a family of intracellular, non-receptor tyrosine kinases including four family members such as JAK1, JAK2, JAK3, and Tyk2. JAK signaling pathways are required for cytokine and growth factor signaling [11]. The downstream molecules, which belong to the signal transducer and activator of transcription (STAT) family, are activated by JAKs. The JAK/STAT pathway is responsible for various functions. JAK2 plays an important role in the signaling pathways induced by hematopoietic growth factors such as granulocyte macrophage colony-stimulating factor (GM-CSF) and interleukin (IL)-3 [12,13]. Although JAK2 is phosphorylated in cytokine-independent BCR-ABL-positive CML cells, aberrant JAK/STAT signaling has been implicated in various tumors [14]. For example, a point mutation in *JAK2* that results in non-synonymous amino acid substitution, V617F, was discovered in hematological malignancies. In fact, the V617F variant is common in patients with myeloproliferative neoplasms (MPNs) such as polycythemia vera, essential thrombocythemia, and primary myelofibrosis [15]. Several JAK2 inhibitors have been developed for patients with MPNs. These inhibitors are currently in clinical trials. One of the JAK2 inhibitors, TG101348 (also known as SAR302503), is a small-molecule JAK2 antagonist. TG101348 inhibits the growth of hematopoietic cells derived from patients with MPNs who have the V617F mutation [16].

JAK2 is part of the BCR-ABL signaling network pathway and is activated in CML cells [17]. JAK2 including the point mutation is also involved in CML maintenance [18-20]. Thus, JAK2 inhibitors may become a therapeutic target for CML cells. Although several reports have demonstrated that BCR-ABL/JAK2 inhibits CML cells including ABL TKI-resistant cells [21,22], it is not completely known whether JAK2 is involved in CML stem cell survival mediated by cytokines in the presence of ABL TKI.

Here, we investigated the effect of TG101348 on residual CML cells. We demonstrated that co-treatment with imatinib and TG101348 increased the cytotoxic effect in CD34-positive CML samples. We also found that cytokine production, which supported growth of CML cells, was reduced by TG101348.

## Results

### Effects of imatinib on BCR-ABL-expressing cells in the presence of human stromal cells

**Figure 1:**
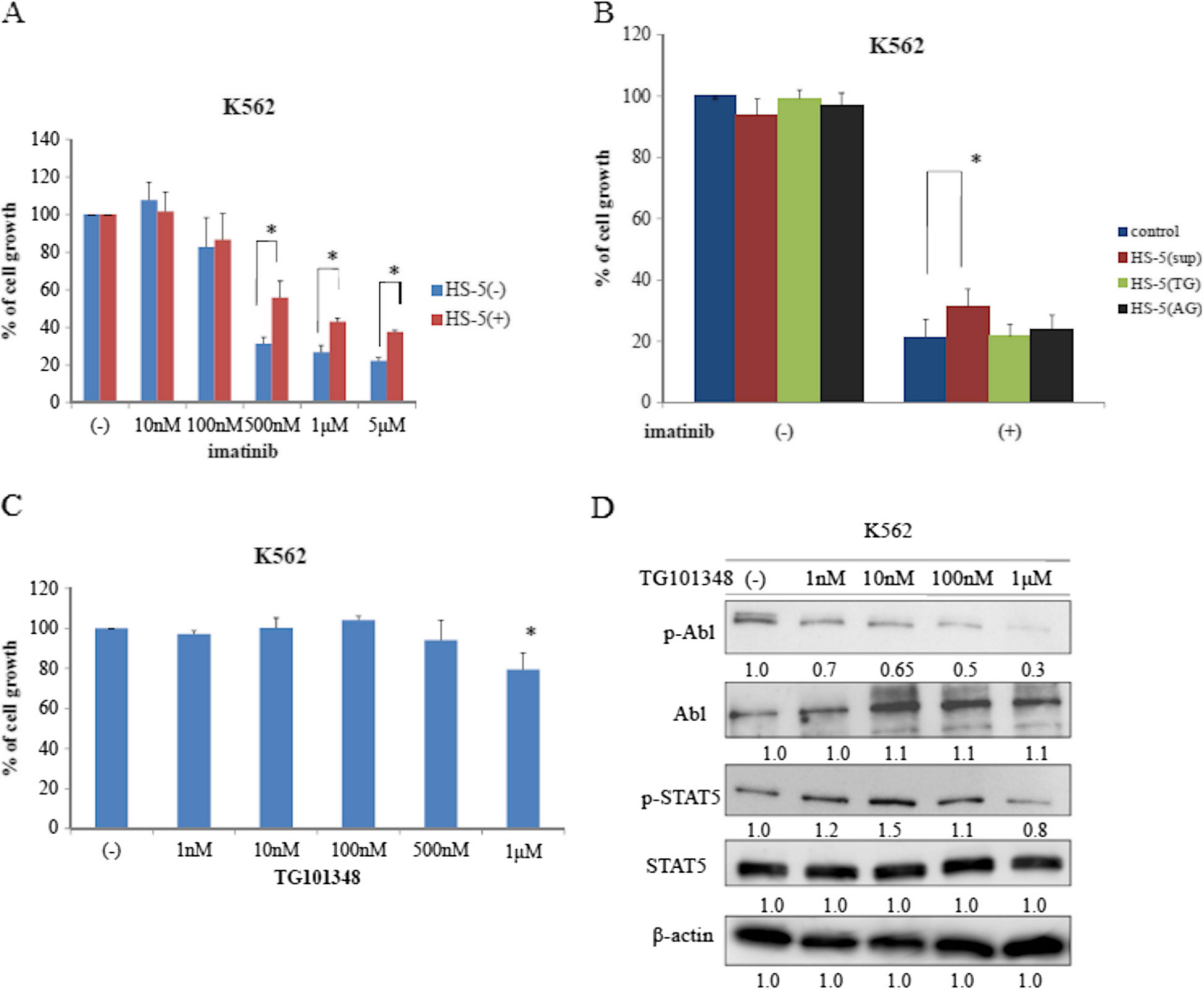
**Cytotoxic effects of imatinib in the presence of HS-5 conditioned medium. (A)** K562 cells were cultured at a concentration of 8 × 10^4^/mL in the presence of varying concentrations of imatinib in the presence or absence of HS-5 conditioned medium for 72 h. Viable cell numbers were calculated. Results are representative of three separate experiments. **(B)** K562 cells were treated with 2 μM imatinib alone or in combination with either 1 μM TG101348 or 5 μM AG490 in the presence or absence of HS-5 conditioned medium for 72 h. Viable cell numbers were calculated. Results are representative of three separate experiments. **(C)** K562 cells were cultured with the indicated concentrations of TG101348 for 72 h. Viable cell numbers were calculated. Results are representative of three separate experiments. **(D)** K562 cells were treated with TG101348 for 24 h, and total extracts were examined by immunoblotting using anti-phospho ABL, ABL, phospho-STAT5, STAT5, and β-actin Abs. Blots were scanned and optical densities were determined using ImageJ software.

We investigated the cell proliferation effects of imatinib on K562 cells when cultured in the presence or absence of HS-5 conditioned medium, which was collected and pooled from a HS-5 stromal cell culture. We found that K562 cell proliferation was inhibited by imatinib in a dose-dependent manner when cultured in the absence of HS-5 conditioned medium (Figure 1A). In contrast, we observed that anti-leukemic activity of imatinib was partially reduced in the presence of HS-5 conditioned medium (Figure 1A). The HS-5 stromal cell line secretes several cytokines [23]. As JAK2 is essential for signaling of several of these cytokines, we used the JAK2 inhibitor TG101348 to investigate the role of JAK2 in the observed protection of K562 cells by HS-5 conditioned medium. We found that co-treatment with imatinib and TG101348 inhibited K562 cell proliferation in the presence of the HS-5 conditioned medium (Figure 1B). We also found that another JAK inhibitor, AG490, also inhibited K562 cell growth in the presence of HS-5 conditioned medium (Figure 1B). We next investigated the effect of TG101348 alone on K562 cells. We found that high TG101348 concentration partially inhibited K562 cell proliferation in the absence of the HS-5 conditioned medium (Figure 1C). The IC50 value for TG101348 was up to 2 μM in BCR-ABL-positive cells. The concentration of TG101348 used in a clinical trial was >1 μM [16]. It has been reported that a high TG101348 concentration is associated with severe adverse events in patients with MF [16], thus, we investigated concentrations below <1 μM in this study. Next, we investigated the effects of this inhibitor on intracellular signaling. We observed a decrease in BCR-ABL and STAT5 phosphorylation in the presence of a high TG101348 concentration (Figure 1D).

### Treatment of Ph-positive (Ph+) leukemia cells with imatinib and TG101348

**Figure 2:**
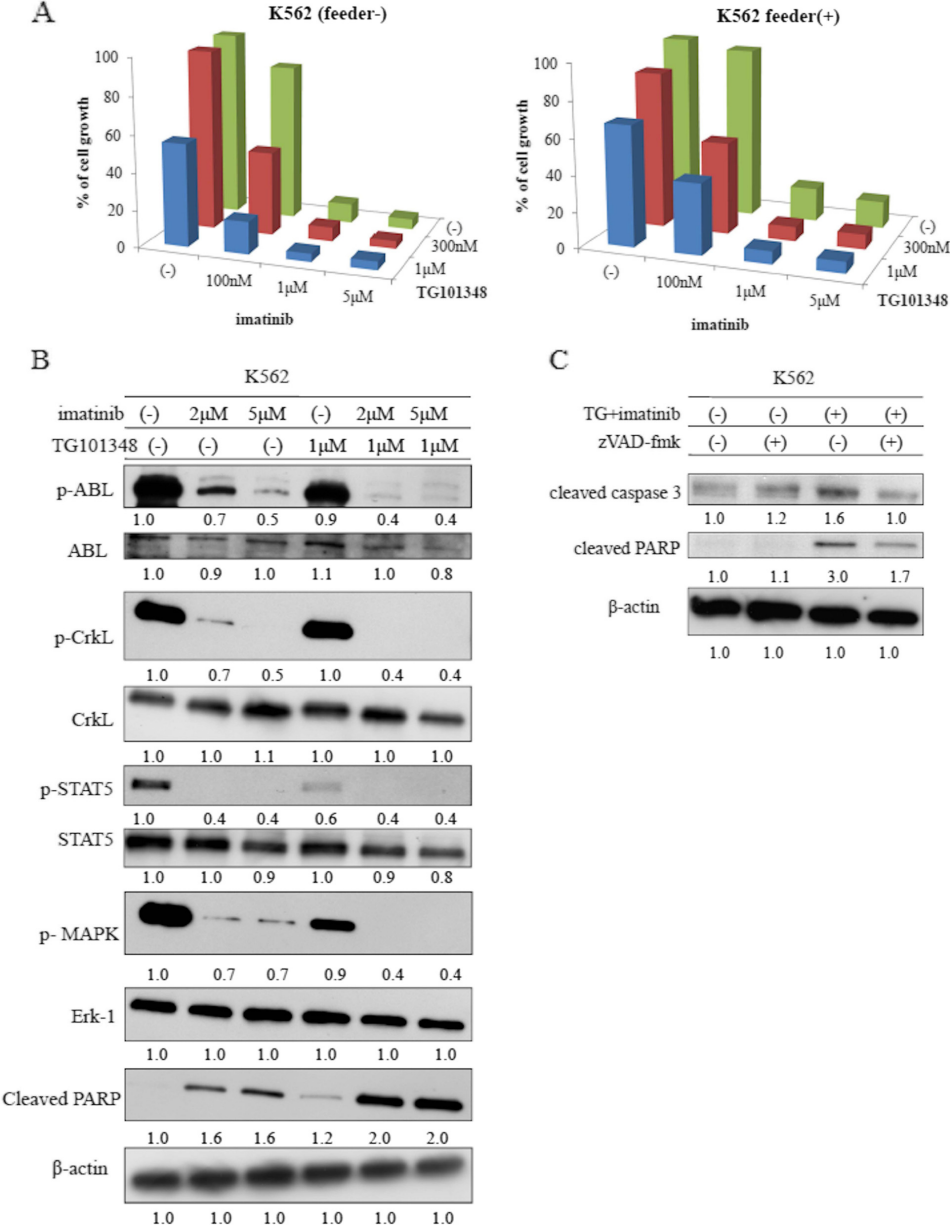
**Co-treatment with imatinib and TG101348 increased inhibition of cell growth and induced apoptosis of BCR-ABL-expressing cells in the presence of HS-5 cells. (A)** K562 cells were cultured at a concentration of 8 × 10^4^/mL in the presence or absence of HS-5 cells. Cells were then treated with varying concentrations of imatinib alone or in combination with TG101348 for 72 h. Viable cell numbers were calculated. Results are representative of three separate experiments. **(B)** K562 cells were treated with imatinib alone or in combination with TG101348 for 24 h, and total extracts were examined by immunoblotting using anti-phospho ABL, ABL, phospho-Crk-L, Crk-L, phospho-STAT5, STAT5, phospho-MAPK, Erk-1, cleaved PARP, and β-actin Abs. Blots were scanned and optical densities were determined using ImageJ software. **(C)** K562 cells were cultured with Z-VAD-fmk (20 μM) and/or TG101348 and imatinib for 24 h. Total extracts were analyzed by immunoblotting using anti-cleaved caspase-3 and cleaved PARP Abs. Actin was used as the loading control. Data are representative of two separate experiments. Blots were scanned and optical densities were determined using ImageJ software.

We next investigated whether imatinib and TG101348 could inhibit Ph + cell growth in the presence of HS-5 cells. K562 cells were exposed to imatinib alone or in combination with TG101348 at different concentrations in the presence or absence of HS-5 cells. Growth of K562 cells treated with imatinib alone in the absence of HS-5 was considerably inhibited in a dose-dependent manner. However, when K562 cells were treated in the presence of HS-5 cells, the effects of imatinib decreased considerably (Figure 2A). Thus, HS-5 cells supported K562 cell proliferation even in the presence of a high imatinib concentration. Next, we investigated the effect of TG101348 treatment. We observed that cell contact between K652 and HS-5 cells was required for protection from imatinib. We also found that treatment with TG101348 overcame the HS-5-mediated protection of K562 cells (Figure 2A). Using immunoblotting, we determined that phosphorylation of Crk-L, a BCR-ABL substrate, STAT5, and mitogen activated protein kinase (MAPK) decreased following co-treatment with imatinib and TG101348. In contrast, we observed that poly(ADP-ribose) polymerase (PARP) activity increased during co-treatment (Figure 2B). Next, we used the pan-caspase inhibitor Z-VAD-fmk to inhibit caspase pathways. We found that Z-VAD-fmk treatment inhibited imatinib- and TG101348-induced apoptosis, suggesting that a caspase-dependent mechanism is involved in TG101348- and imatinib-mediated cell death in K562 cells (Figure 2C).

### Effects of TG101348 in combination with imatinib on CD34-positive CML samples

**Figure 3:**
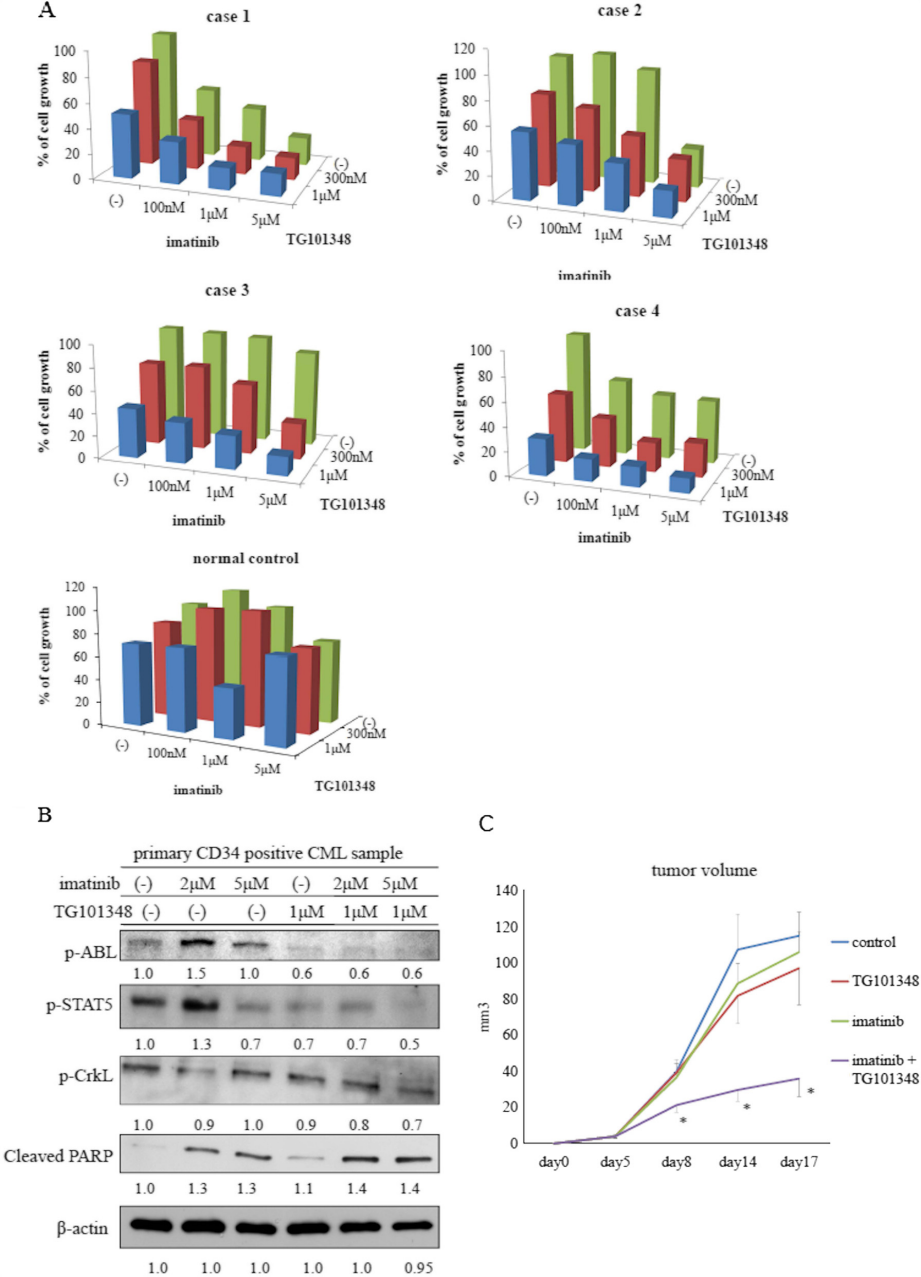
**Effects of imatinib and TG101348 on CD34-positive primary cells. (A)** CD34-positive CML cells or healthy donor samples were cultured in the presence of HS-5 cells. Cells were then treated with varying concentrations of imatinib alone or in combination with TG101348 for 72 h. Viable cell numbers were calculated. Results are representative of three separate experiments. **(B)** CD34-positive primary cells were treated with the indicated concentrations of imatinib alone or in combination with TG101348 for 24 h. Total cell extracts were analyzed by immunoblotting using phospho-specific ABL, STAT5, Crk-L, and cleaved PARP Abs. β-actin was used as the loading control. Results are representative of at least three reproducible experiments. Blots were scanned and optical densities were determined using ImageJ software. **(C)***In vivo* studies were performed as described in Methods. Tumors were treated with or without imatinib and TG101348 5 days per week, and growth was measured. *p < 0.05, compared with control mice.

To determine whether co-treatment with TG101348 and imatinib affects primary chronic phase CML cell viability, we isolated untreated peripheral blood samples from patients at the time of diagnosis or healthy donors and cultured the cells in the presence of HS-5 cells for 72 h with either imatinib alone or in combination with TG101348 at the indicated concentrations (Figure 3A). Cell viability was assessed by trypan blue exclusion. Viability of CD34-positive primary cells was not affected by imatinib treatment alone or in the presence of HS-5 stromal cells (Figure 3A). In contrast, viability of the primary cells decreased significantly by imatinib in the absence of HS-5 cells (data not shown). However, co-treatment with TG101348 and imatinib resulted in a progressive reduction in viable cell number compared with treatment with imatinib alone (Figure 3A). Furthermore, viable cell numbers of normal samples decreased partially after treatment with imatinib and TG101348 suggests that these treatments were selectively, and more effectively influence against leukemia (Figure 3A). The efficacy of the co-treatment was different in each group of primary samples, which may indicate different chemical sensitivities of these compounds. We next investigated the effect of the inhibitors on intracellular signaling in CD34-positive primary samples. We found that BCR-ABL, Crk-L, and STAT5 phosphorylation decreased, while PARP activity increased after the co-treatment (Figure 3B). We investigated the efficacy of co-treatment with TG101348 and imatinib in a xenograft model. Growth of tumors decreased considerably in TG101348- and imatinib-treated mice compared with that in control mice. These results indicate that co-treatment with imatinib and TG101348 is an effective strategy to reduce expansion of BCR-ABL-positive cells including CD34-positive primary cells.

### Knockdown of JAK2 increased sensitivity to imatinib

**Figure 4:**
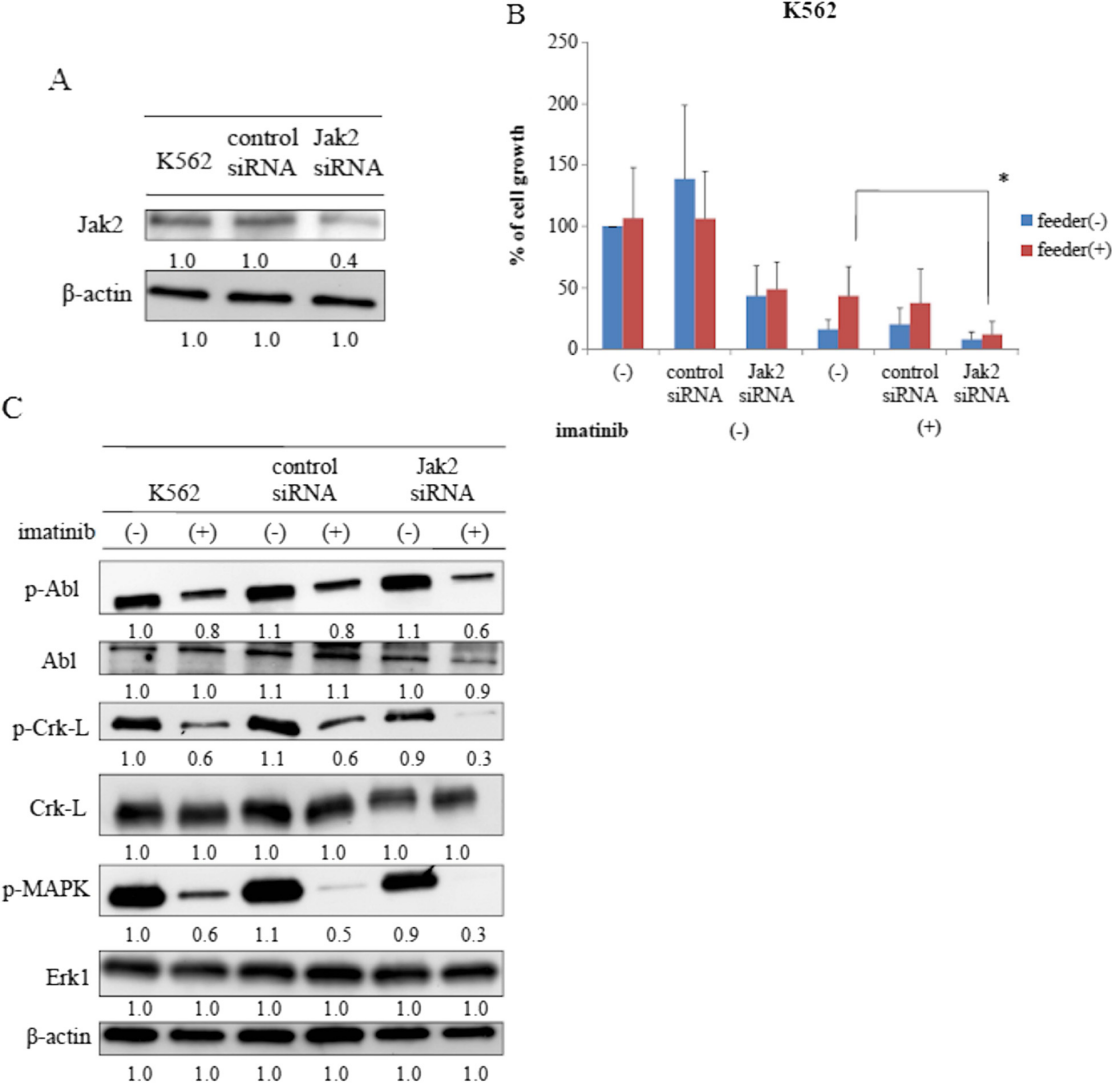
**JAK2 siRNA transfected K562 cells had increased sensitivity to imatinib. (A)** K562 cells were transfected with JAK2 siRNA or a control siRNA. Cell lysates were analyzed by immunoblotting 72 h after transfection using Jak2 and β-actin Abs. Blots were scanned and optical densities were determined using the ImageJ system. **(B)** K562 and siRNA-transfected K562 cells were cultured at a concentration of 8 × 10^4^/mL in the presence or absence of feeder cells. The cells were treated with 1 μM imatinib for 72 h. Viable cell numbers were calculated. Results are representative of three separate experiments. **(C)** K562 and siRNA-transfected K562 cells were treated with imatinib for 24 h, and total extracts were examined by immunoblotting using anti-phospho ABL, ABL, phospho-Crk-L, Crk-L, phospho-MAPK, Erk-1 and β-actin Abs. Blots were scanned and optical densities were determined using ImageJ software.

We next knocked down JAK2 by transfecting JAK2 small interfering RNA (siRNA) into K562 cells. Cell lysates were analyzed by immunoblotting 72 h after transfection using an anti-JAK2 Ab. We confirmed that Jak2 expression decreased considerably when compared to that in control cells (Figure 4A). To test the effect of JAK2 knockdown on K562 cells, we examined the effect of imatinib on them when cultured in the presence of HS-5. We observed that the viability of JAK2 siRNA transfected cells was not reduced when compared with that of control cells (Figure 4B). In contrast, viability of JAK2 siRNA-transfected cells was partially reduced in the presence of HS-5 cells when compared to control cells cultured under the same conditions (Figure 4B). Importantly, we found that viability of JAK2 siRNA-transfected K562 cells decreased considerably after imatinib treatment in the presence of HS-5 cells (Figure 4B). Next, we investigated the effect of imatinib on intracellular signaling in these cells. We found that BCR-ABL phosphorylation in the JAK2 siRNA-transfected K562 cells was not reduced after imatinib treatment compared to control cells (Figure 4C). In contrast, we observed that Crk-L and MAPK phosphorylation decreased after imatinib treatment in JAK2-siRNA transfected K562 cells (Figure 4C). Thus, these results indicate that JAK2 is involved in the downstream signaling of BCR-ABL.

### Detection of cytokines in HS-5 conditioned medium

**Figure 5:**
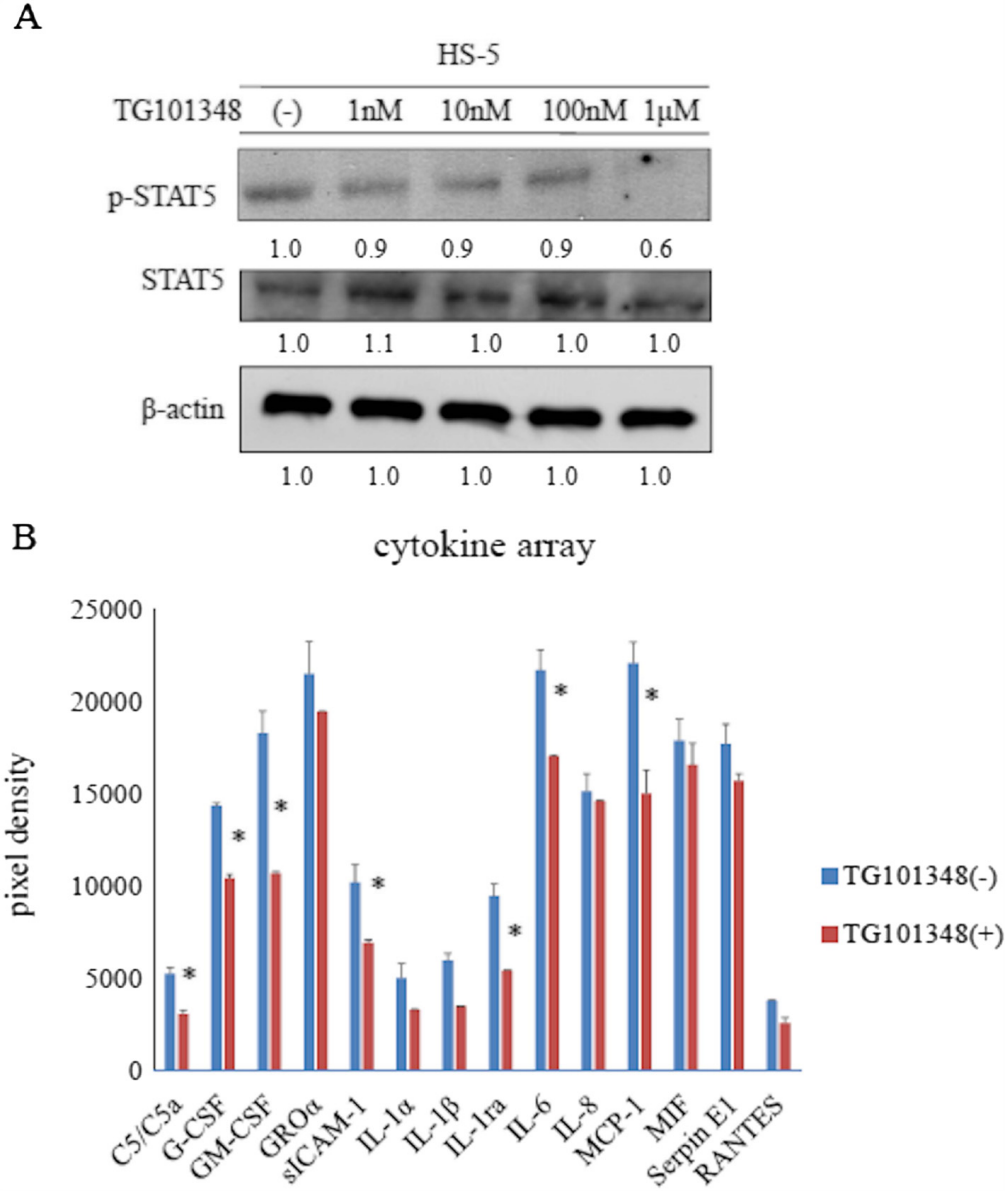
**Effect of TG101348 on HS-5 cells. (A)** HS-5 cells were cultured in the presence of different concentrations of TG101348 for 24 h, and total extracts were examined by immunoblotting using anti-phospho STAT5, STAT5, and β-actin Abs. Blots were scanned and optical densities were determined using ImageJ software. **(B)** HS-5 cells were treated with TG101348 for 24 h and conditioned medium was collected. The relative levels of 36 different cytokines were analyzed using the Human Cytokine Array Kit. The expression of cytokines was determined using ImageJ software. Results are representative of three separate experiments. *p < 0.05, TG101348 treatment vs. control.

The HS-5 cell line was treated with TG101348 at different concentrations for 24 h (Figure 5). We found that STAT5 phosphorylation was partially reduced after TG101348 treatment using immunoblotting analysis (Figure 5A). We next investigated cytokine levels by assaying HS-5 conditioned medium after 1 day of culture in the presence or absence of TG101348 using the human cytokine array according to the manufacturer’s instructions. We analyzed 36 cytokines simultaneously and evaluated their relative expression levels. We found that GM-CSF, and IL-6 levels decreased considerably after TG101348 treatment compared to that in the untreated conditioned medium (Figure 5B). These results indicate that TG101348 regulates cytokine production in HS-5 cells.

## Discussion

Previous studies revealed that BCR-ABL-positive progenitor cells persist in patients with CML despite TKI therapy. This may be attributable, in part, to quiescent CML stem cells residing in protected hematopoietic niches [24]. Hematopoietic growth factors, including cytokines, are secreted by endothelial cells and fibroblasts. JAK2 phosphorylates STAT5 in normal hematopoietic stem cells following activation of cytokine receptors. However, BCR-ABL phosphorylates STAT5 at the same critical tyrosine residue close to the SH2 domain in CML cells, and activated STAT5 translocates to the nucleus [14,25].

Residual leukemia cells in bone marrow niches are exposed to many cytokines *in situ*[26]. Our results indicate that cytokine-enriched HS-5 conditioned medium protected CML cells from imatinib. Furthermore, we demonstrated that the imatinib inhibitory effect decreased when CML cells were co-cultured with HS-5 cells. This stromal cell line secretes multiple cytokines that support hematopoietic progenitor proliferation. Treatment of K562 cells with imatinib in the presence of HS-5 conditioned medium resulted in only partial inhibition of cell growth, indicating that imatinib alone may not be sufficient to eradicate residual CML cells that reside within a cytokine-rich microenvironment. Thus, it is important to probe the cytokine-mediated leukemia stem cell signaling pathways as targets for combined treatments with TKIs to develop more efficient therapies for patients with CML.

In this study, we analyzed the ability of cytokine signaling to protect CML cells. A previous study revealed GM-CSF-mediated imatinib and nilotinib resistance through BCR-ABL independent activation of the JAK2/STAT5 pathway [27]. We demonstrated that inhibiting JAK2 by either JAK2 siRNA transfection or the JAK2 inhibitor TG101348 downregulated STAT5 activity. Moreover, STAT5 phosphorylation decreased considerably and PARP activity increased following co-treatment with imatinib and TG101348 in Ph^+^ leukemia cells including primary CD34-positive cells. Inhibiting STAT5 in leukemia was sufficient to prevent leukemia cell proliferation. JAK2 functions downstream of BCR-ABL. Although we did not tried to combine the two drugs at their IC50s in this cell line, we showed that inhibiting JAK2 resulted in reduced STAT5 activity and enhanced imatinib efficacy even when leukemia cells were protected by hematopoietic stromal cells-mediating cytokine/chemokine production.

We also examined the effect of TG101348 on cytokine expression in HS-5 cells using a cytokine array containing 36 different cytokines. We observed that expression levels of several cytokines, such as GM-CSF, G-CSF, and IL-6, decreased considerably after treatment. Because GM-CSF and IL-6 are involved in maintenance of hematopoietic stem cells, these cytokines suppress apoptosis and regulate cell viability, growth, and differentiation [28]. Thus, activating cytokine-mediated JAK2/STAT5 signaling may circumvent the need for BCR-ABL signaling and cell survival [29]. Here, we demonstrated that blocking cytokine signaling using a JAK2 inhibitor in combination with imatinib might be an effective strategy to eradicate residual CML cells *in vitro* and *in vivo*. Cytokine-mediated signaling may not be critical for cell survival when BCR-ABL signaling is activated; however, it is possible that residual CML cells treated with imatinib can be rescued through a cytokine-triggered JAK2/STAT5 signaling pathway.

## Conclusions

Our results indicate that co-treatment with imatinib and JAK2 inhibitors may have the potential for targeting residual CML cells by enhancing imatinib efficiency and reducing cytokine production by stromal cells. Therefore, this approach may represent an important therapeutic strategy for patients with CML.

## Methods

### Cell culture and reagents

The BCR-ABL-positive leukemia cell line K562 and human bone marrow stromal cell line HS-5 were obtained from the American Type Culture Collection (Rockville, MD, USA). Cells were cultured in the Roswell Park Memorial Institute 1640 medium containing 10% fetal bovine serum and maintained at 37°C in a 5% CO_2_ humidified atmosphere. The JAK2 inhibitor TG101348 was purchased from Shanghai APIs Chemical (Shanghai, China) and the ABL kinase inhibitor imatinib was provided by Novartis Pharma AG (Basel, Switzerland). Stock solutions of TG101348 were prepared in dimethyl sulfoxide, while that of imatinib was prepared in distilled water. These stock solutions were further diluted in growth medium to achieve the desired concentrations. Antibodies (Abs) against phosphor-Abl, phospho Crk-L, STAT5, MAPK, cleaved caspase-3, and PARP were purchased from Cell Signaling Technology (Danvers, MA, USA). Erk-1, Crk-L, and STAT5 Abs were purchased from BD Biosciences (San Jose, CA, USA). The anti-Abl Ab was purchased from Santa Cruz Biotechnology (Santa Cruz, CA, USA). Other reagents were obtained from Sigma-Aldrich (St. Louis, MO, USA).

### Primary BCR-ABL-positive and normal control cells

This study protocol was approved by the Institutional Review Board of Tokyo Medical University, and written informed consent was provided by all patients in accordance with the Declaration of Helsinki. Primary samples were collected from untreated peripheral blood of patients with CML or from the bone marrow of normal healthy donor after obtaining written informed consent. The peripheral blood was collected in heparinized tubes, and mononuclear cells were separated using LymphoSepare (Immuno-Biological Laboratories, Minneapolis, MN, USA). These cells were either used immediately or cryopreserved in liquid nitrogen until use.

### Cell proliferation assay

Cells were seeded in 24- or 96-well plates at a density of 8 × 10^4^ or 8 × 10^3^ cells/well. Next, cells were treated with the inhibitors at the indicated concentrations: imatinib, 100 nM to 2 μM; and TG101348, 100 nM to 1 μM. Viable cells were either counted using trypan blue exclusion or were stained with a cell counting kit solution (Dojin, Kumamoto, Japan) and measured spectrophotometrically (A_450_) to determine cell viability. In some experiments, cells were co-cultured with human stromal cells. After cultivating the HS-5 cells at a density of 1 × 10^5^ cells/well in 24-well plates overnight, the K562 cells were plated onto the HS-5 cells, cultured for 3 days, and analyzed for cell proliferation. To analyze the activity of the soluble factors produced by HS-5 cells, K562 cells were cultured for 72 h in the presence of HS-5 conditioned medium from cultures that that were either not treated with imatinib or treated with the drug at the indicated concentrations. Cell proliferation was then analyzed.

### siRNA transfection

siRNAs targeting the JAK family members were purchased from Santa Cruz Biotechnology (sc-39099). K562 cells were transfected with a siRNA targeting JAK2 by electroporation, as described previously [30].

### Immunoblotting

Immunoblotting was performed as described previously [31]. After different treatments were performed as indicated above, cells were collected by centrifugation and lysed by sonication using the radioimmunoprecipitation assay lysis buffer. Protein content in the lysates was determined using a protein assay kit (Bio-Rad Laboratories, Hercules, CA, USA). Forty micrograms of total cellular proteins were separated on 4–20% polyacrylamide gels and transferred to polyvinylidene difluoride membranes. Next, the membranes were probed using primary Abs of interest at the appropriate dilutions for 2 h at room temperature. The blots were visualized by the chemiluminescent method using the Amersham ECL chemiluminescence kit (GE Healthcare, Tokyo, Japan). Immunoblots were quantified using ImageJ software (NIH, Bethesda, MD, USA).

### Cytokine array

After culturing HS-5 cells in the presence or absence of TG101348, the culture medium was collected. Cytokine levels in the medium were measured using the human cytokine panel A (Proteome Profiler™) (R&D Systems, Minneapolis, MN, USA). Horseradish peroxidase substrate was used to detect protein expression, and data were visualized using Kodak Biomax Light film. Arrays were scanned and optical densities were determined using ImageJ software.

### In vivo studies

Six-week-old female BALB/c nude mice were purchased from CLEA Japan Inc. (Tokyo, Japan). Mice were injected subcutaneously with 1 × 10^7^ Ba/F3 wild-type BCR-ABL cells. Mice were treated after 5 days with 40 mg/kg imatinib intraperitoneally or 30 mg/kg TG101348 orally or a combination of the two agents for 5 days per week. Control mice were treated with phosphate-buffered saline. Tumor sizes were analyzed every day. The average tumor weight per mouse (n = 6) was calculated.

### Statistical analysis

We used Student’s *t*-test to determine differences in response to the various treatments. *p* < 0.05 was considered statistically significant.

## Abbreviations

Ph: Philadelphia chromosome; TKI: Tyrosine kinase inhibitor; CML: Chronic myeloid leukemia; JAK: Janus kinase; GM-CSF: Granulocyte macrophage colony-stimulating factor; STAT: Signal transducer and activator of transcription; IL: Interleukin.

## Competing interests

The authors declare no competing interests.

## Authors’ contributions

SO, TT, SK, YT, and KO conceived and designed the experiments; SO performed the experiments; and SO, TT, SK, YT, and KO contributed reagents and wrote the manuscript. All authors read and approved the final manuscript.
